# Mucopolysaccharidosis type II: European recommendations for the diagnosis and multidisciplinary management of a rare disease

**DOI:** 10.1186/1750-1172-6-72

**Published:** 2011-11-07

**Authors:** Maurizio Scarpa, Zsuzsanna Almássy, Michael Beck, Olaf Bodamer, Iain A Bruce, Linda De Meirleir, Nathalie Guffon, Encarna Guillén-Navarro, Pauline Hensman, Simon Jones, Wolfgang Kamin, Christoph Kampmann, Christina Lampe, Christine A Lavery, Elisa Leão Teles, Bianca Link, Allan M Lund, Gunilla Malm, Susanne Pitz, Michael Rothera, Catherine Stewart, Anna Tylki-Szymańska, Ans van der Ploeg, Robert Walker, Jiri Zeman, James E Wraith

**Affiliations:** 1 Department of Pediatrics, University of Padua, Padua, Italy; 2 Department of Pediatrics, Heim Pál Hospital for Sick Children, Budapest, Hungary; 3 University Medical Center, Johannes Gutenberg University, Mainz, Germany; 4 Department of Human Genetics, Miller School of Medicine, Miami, FL, USA; 5 Department of Paediatric Otorhinolaryngology, Royal Manchester Children's Hospital, Manchester, UK; 6 Department of Pediatric Neurology and Metabolic Diseases, UZ Brussel, Brussels, Belgium; 7 Reference Centre of Metabolic Diseases, HFME Hospital, Lyon, France; 8 Medical Genetics Unit, Department of Pediatrics, University Hospital Virgen de la Arrixaca, El Palmar, Murcia, Spain; 9 Therapy and Dietetics Department, Royal Manchester Children's Hospital, Manchester, UK; 10 Manchester Academic Health Science Centre, University of Manchester, Central Manchester University Hospitals NHS Foundation Trust, Manchester, UK; 11 Department of Pediatrics, Children's Hospital and Johannes Gutenberg University, Mainz, Germany; 12 Klinik für Kinder- und Jugendmedizin am Evangelischen Krankenhaus Hamm, Hamm, Germany; 13 Division of Cardiology and Division of Lysosomal Storage Diseases, University Children's Hospital, Mainz, Germany; 14 Children's Hospital, University Medical Center, Johannes Gutenberg University, Mainz, Germany; 15 Society for Mucopolysaccharide Diseases, Amersham, UK; 16 Unidade Doenças Metabólicas, Serviço de Pediatria, Hospital de S. João, Porto, Portugal; 17 Division of Metabolism, Connective Tissue Unit, University Children's Hospital, Zurich, Switzerland; 18 Department of Clinical Genetics, Rigshospitalet, Copenhagen University Hospital, Denmark; 19 Department of Pediatrics, Karolinska Institute, Stockholm, Sweden; 20 Department of Ophthalmology, University Medical Center, Johannes Gutenberg University, Mainz, Germany; 21 Birmingham Children's Hospital, Birmingham, UK; 22 The Children's Memorial Health Institute, Clinic of Metabolic Diseases, Warsaw, Poland; 23 Department of Pediatrics, Center for Lysosomal and Metabolic Diseases, Erasmus Medical Center, University Hospital, Rotterdam, The Netherlands; 24 Royal Manchester Children's Hospital, Manchester, UK; 25 Department of Pediatrics and Adolescent Medicine, First Faculty of Medicine, Charles University and General University Hospital, Prague, Czech Republic

## Abstract

Mucopolysaccharidosis type II (MPS II) is a rare, life-limiting, X-linked recessive disease characterised by deficiency of the lysosomal enzyme iduronate-2-sulfatase. Consequent accumulation of glycosaminoglycans leads to pathological changes in multiple body systems. Age at onset, signs and symptoms, and disease progression are heterogeneous, and patients may present with many different manifestations to a wide range of specialists. Expertise in diagnosing and managing MPS II varies widely between countries, and substantial delays between disease onset and diagnosis can occur. In recent years, disease-specific treatments such as enzyme replacement therapy and stem cell transplantation have helped to address the underlying enzyme deficiency in patients with MPS II. However, the multisystem nature of this disorder and the irreversibility of some manifestations mean that most patients require substantial medical support from many different specialists, even if they are receiving treatment. This article presents an overview of how to recognise, diagnose, and care for patients with MPS II. Particular focus is given to the multidisciplinary nature of patient management, which requires input from paediatricians, specialist nurses, otorhinolaryngologists, orthopaedic surgeons, ophthalmologists, cardiologists, pneumologists, anaesthesiologists, neurologists, physiotherapists, occupational therapists, speech therapists, psychologists, social workers, homecare companies and patient societies.

**Take-home message:**

Expertise in recognising and treating patients with MPS II varies widely between countries. This article presents pan-European recommendations for the diagnosis and management of this life-limiting disease.

## Introduction

Mucopolysaccharidosis type II (MPS II, Hunter syndrome, Online Mendelian Inheritance in Man number 309900) is an X-linked, recessive disease that is characterised by deficiency in the activity of the lysosomal enzyme iduronate-2-sulfatase (I2S), owing to a mutation in the I2S gene (*IDS*) [1,2]. Like other mucopolysaccharidoses, the enzyme deficiency in MPS II results in the lysosomal accumulation of glycosaminoglycans (GAGs). The condition is multisystem in nature, with patients exhibiting coarsening of facial features, bone and joint abnormalities, short stature, and changes in the heart, respiratory system, hearing, and vision [2]. Severely affected patients have profound neurological involvement, with progressive learning difficulties and behavioural abnormalities, as well as disturbed motor function [3].

MPS II is one of the most common mucopolysaccharidoses, with an estimated prevalence of 1 in 140 000-156 000 live births in Europe [4-6]. The disease affects males almost exclusively, although a few symptomatic females have been identified [7-10]. Age at onset and disease progression are heterogeneous: patients typically have a normal appearance at birth, with the initial signs and symptoms emerging between the ages of 18 months and 4 years, depending on disease severity [2,11,12]. Because the initial signs and symptoms of MPS II can be non-specific, identification of patients at a young age can be problematic, resulting in a substantial delay between disease onset and diagnosis. Life expectancy varies according to disease severity; patients with severe phenotypes are expected to live for less than 2 decades, whereas individuals with attenuated forms of MPS II may survive into their 50s or 60s [2,3,13].

Until recently, the management of patients with MPS II has been largely supportive, focussing on the treatment of signs and symptoms rather than addressing the underlying lysosomal enzyme deficiency. Disease-specific therapy for MPS II is now available throughout Europe, although expertise in diagnosis and managing MPS II varies widely between countries. Thus, there is a need for guidance on how to recognise, diagnose and manage patients with this condition, with particular focus given to the multidisciplinary approach needed for this multisystem disease.

This article describes the recommendations developed by the Hunter Syndrome European Expert Council (HSEEC) for the diagnosis and management of MPS II. The HSEEC is a group of European clinicians with substantial experience of diagnosing and treating patients with MPS disorders and lysosomal storage diseases (LSDs). Others with expertise in particular aspects of the management of MPS II have also contributed to these recommendations - including specialist clinicians, a specialist nurse and a representative of a patient society - with the aim of providing practical guidance on all aspects of patient care. A full list of contributors can be found at the front of this article.

## Methodology

These recommendations have been developed using an evidence-based approach. Owing to the rarity of MPS II, there is a paucity of published data on the management of this disease, so data from clinical trials, observational studies, review articles and case studies were all considered when formulating recommendations. For those topics for which few or no published data were available, the information presented is based on the clinical experience of the authors. Such instances are clearly indicated in the manuscript as Consensus Opinion (CO).

### Search strategy and selection criteria

Literature searches for topics relating to the management of patients with MPS II were carried out in PubMed and EMBASE between 13 July and 16 September 2010, using Medical Subject Heading Terms and relevant keywords. To ensure relevance to the modern day clinical setting, literature searches were limited to articles published since 1 January 1990. Older articles identified by the authors were also included. Only articles from the peer-reviewed literature were included in the literature search. Articles in a non-English language with an abstract, and articles in the English language without an abstract were included if they were considered relevant to the search being carried out. Abstracts from industry-sponsored meetings were not included.

## Diagnosis

**Figure 1:**
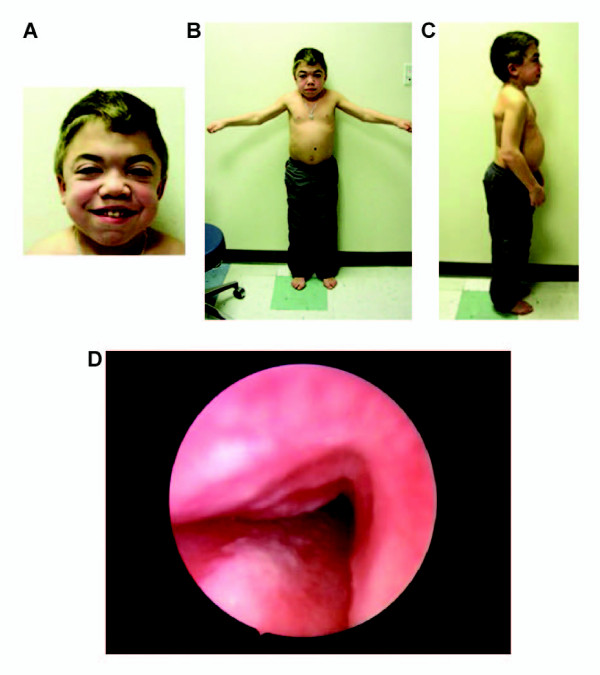
**Characteristic features of mucopolysaccharidosis type II**. Fourteen-year-old boy showing **(a) **coarsened facial features (including enlarged head, broad nose with flared nostrils, prominent supraorbital ridges, large jowls, thickened lips, and irregular peg-shaped teeth), **(b) **musculoskeletal manifestations (including short neck, short stature, and joint stiffness [unable to raise arms above head]) and **(c) **abdominal distension due to hepatomegaly and splenomegaly. **(d) **Tracheomalacia seen at airway endoscopy. (**a)-(b) **reproduced with permission from Martin and colleagues [11], Copyright ^© ^2008 by the AAP; **(a)-(c) **courtesy of Professor Joseph Muenzer; **(d) **courtesy of Dr Iain Bruce.

MPS II is a progressive disorder that has traditionally been categorised into a severe form and a mild/attenuated form based on the age at onset of signs and symptoms, the presence or absence of neurological involvement, and length of survival [2]. However, this classification appears to be a gross oversimplification, particularly as I2S activity is equally deficient in both forms of the disease [2]. Rather, the disorder should be regarded as a continuum of phenotypes between two extremes [3]. The multisystem nature of MPS II and the heterogeneity of disease progression mean that patients may present with many different signs and symptoms to a wide range of specialists. Characteristic features of MPS II include coarsened facial features, an enlarged head, an enlarged tongue, hypertrophic tonsils and adenoids, irregularly shaped teeth, recurrent otitis media, a distended abdomen due to hepatosplenomegaly, abdominal and/or inguinal hernias, and thickened pebbled skin (Figure 1) [11,14]. Patients with MPS II also exhibit short stature, although children with severe phenotypes are often initially taller than their peers, before growth slows [12]. Other signs and symptoms can result from changes to the musculoskeletal system, eyes, gastrointestinal tract, airways and cardiovascular and nervous systems. Although the specific combination of signs and symptoms may vary considerably between individuals, the presence of any of the features listed may be suggestive of MPS II. The evolution of signs and symptoms is often a better indicator of a diagnosis of MPS II than a static snapshot of the presence or absence of certain manifestations. Therefore, it is important to monitor changes in signs and symptoms over time (CO).

**Figure 2:**
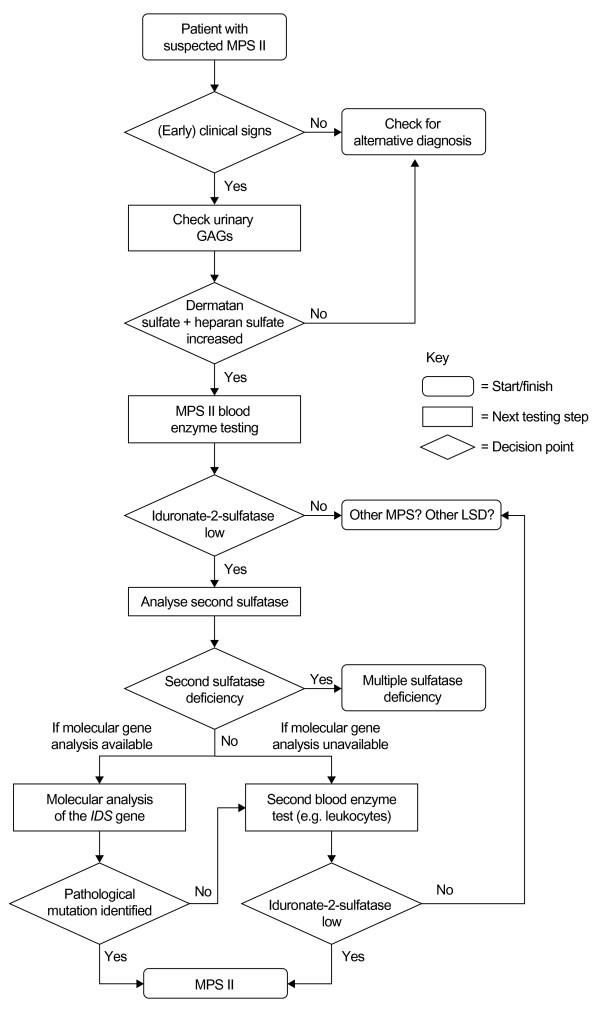
**Diagnostic algorithm for mucopolysaccharidosis type II (MPS II)**. The 'gold standard' for the diagnosis of MPS II in a male proband is demonstration of deficiency of iduronate-2-sulfatase enzyme activity in leukocytes, fibroblasts, or plasma. Measurement of iduronate-2-sulfatase activity in dry blood spots also represents a valuable method for diagnosis, as no heparin is needed and very little blood is required. GAGs = glycosaminoglycans. *IDS *= iduronate-2-sulfatase gene. LSD = lysosomal storage disease. MPS = mucopolysaccharidosis. MSD = multiple sulfatase deficiency.

A suspected diagnosis of MPS II should be confirmed by biochemical or genetic analysis. An algorithm for testing for MPS II is shown in Figure 2. Quantitative and qualitative analysis of urinary GAGs is useful as a preliminary screening test to help establish that an individual has a form of MPS; however, this does not confirm a specific diagnosis of MPS II [2]. The 'gold standard' for the diagnosis of MPS II in a male proband is demonstration of deficiency of I2S enzyme activity in leukocytes, fibroblasts, or plasma. Measurement of I2S activity in dry blood spots also represents a valuable method for screening, as no heparin is needed and very little blood is required. Dry blood spots are stable for several days at room temperature so transportation of samples is easy [15], and this may extend testing to areas some distance from diagnostic centres, which are not widely available. Documentation of normal enzyme activity of at least one other sulfatase is critical, as low levels of I2S activity are also characteristic of multiple sulfatase deficiency [11]. Finally, molecular genetic testing of *IDS *to confirm the diagnosis may be useful in male patients with an unusual phenotype or in whom the results of I2S testing are inconclusive [16]. Molecular genetic testing of *IDS *is also important for genetic counselling, especially if there is no known family history of MPS II. Once the disease-causing mutation has been identified, a detailed pedigree analysis should be carried out to identify family members who may be carriers of a disease-causing mutation or at risk of the disease, and genetic counselling should be offered to all family members.

Prenatal diagnosis and pre-implantation genetic diagnosis can be useful for identifying affected embryos in at-risk pregnancies [17]. Although it is beneficial to have identified the disease-causing mutation in the family [18], it is not always necessary for prenatal diagnosis. If the mother has been diagnosed as an obligate carrier through her family history, assays for the I2S enzyme and GAG levels may be conducted on cells from amniotic fluid, in chorionic villus biopsy tissue or cord blood [19,20], allowing early testing and rapid diagnosis of affected foetuses.

Females with MPS II are very rare. They are typically heterozygous for this X-linked disorder and most are asymptomatic 'carriers' with normal or slightly reduced I2S activity [3]. Some heterozygous females have been found to develop signs and symptoms because of structural abnormalities of the × chromosome or skewed inactivation of the paternal × chromosome [7,8,21-25]. There has also been one report of a female patient who was homozygous for a disease-causing point mutation [9]. The most reliable method of diagnosing MPS II in affected females and carriers is to test for a family-specific mutation that has been identified in an affected male relative [3,16]. Sequence analysis of the entire *IDS *coding region may be necessary if a family-specific mutation is not known. An alternative is first to sequence the exons with the highest prevalence of mutations (e.g. exon IX) or search for recurrent mutations (e.g. p.S333L). Complex rearrangements between *IDS *and its pseudogene, *IDS2*, can also be detected by PCR.

## Assessing disease severity

The advent of an effective treatment for MPS II has highlighted the need for a standardised method for monitoring the progression of patients with this disease and their response to therapy. There is currently no standardised severity scoring system for MPS II. Biomarkers such as urinary GAGs and heparin cofactor II-thrombin complex [26] have been proposed, but neither has been found to be a specific marker of disease severity in patients with MPS II [2,27]. Standard functional tests, such as the Pediatric Evaluation of Disability Inventory (PEDI) and a test developed by the Pediatric Orthopedic Society of North America (POSNA) [28,29], may be applicable to patients with MPS II, but this requires confirmation in a relevant population [30]. The validation of a scoring system for such a rare disease as MPS II represents a significant challenge.

In the absence of a suitable severity scoring system for MPS II, it is recommended that patients are monitored closely and undergo a comprehensive physical, biochemical, and behavioural evaluation following diagnosis and at least every 6-12 months thereafter (CO). More frequent assessment may be necessary in patients in whom signs and symptoms are progressing rapidly. Where possible, this should take place at a centre with experience of treating LSDs. Assessments should include evaluation of the musculoskeletal and cardiovascular systems, ears, airways, eyes, skin, nervous system, abdomen and gastrointestinal system, as outlined in Table 1. Many examinations are dependent on patient cooperation, so proper evaluation may be difficult in very young patients or individuals with cognitive dysfunction. It is important to note that the development of many of the individual signs and symptoms of mucopolysaccharidoses is irreversible; therefore, slowing or halting disease progression should be considered an important outcome in patients receiving treatment (CO).

**Table 1:** Close monitoring of patients with mucopolysaccharidosis type II is necessary.

Presenting feature	Assessment method
*General appearance*	
Enlarged head	Clinical examination (including measurement of head circumference),* family history*
Coarse facial features (broad nose with flared nostrils, prominent supraorbital ridges, large jowls, thickened lips)	Clinical examination*
Irregular, peg-shaped teeth	Clinical examination*
Hyperplasic and hypertrophic gingival tissue	Clinical examination*

*Cardiovascular system*	
Right and left ventricular hypertrophy	Echocardiogram,* chest X-ray,* cardiac MRI,† CT scan†
Arrhythmia, irregular heartbeat	Clinical examination,* electrocardiogram,* Holter monitoring†
Heart failure	Echocardiogram,* electrocardiogram,* CT scan,† metabolic or perfusion imaging (positron emission tomography and single photo emission computer tomography)†
Changes to mitral, aortic, tricuspid and pulmonary valves	Echocardiogram,* cardiac MRI†
Hypertension	Clinical examination*

*Nervous system*	
Developmental delay	Medical history (achievement of developmental milestones),* neurobehavioral assessment/cognitive testing,* measurement of intelligence quotient†
Progressive mental impairment (cognitive dysfunction)	Neurobehavioral assessment/cognitive testing,* measurement of intelligence quotient†
Gait disturbance	Evaluation of sitting and standing posture and walking ability (6-minute walk test),* MRI of the brain and cranio-cervical junction†
Seizures	MRI of the brain and cranio-cervical junction*, electroencephalography†
Behavioural disturbances (over activity, obstinacy, aggression)	Neurobehavioral assessment/cognitive testing,* measurement of intelligence quotient†
Carpal tunnel syndrome	Electrophysiological testing of nerve conduction velocity†

*Eye*	
Loss of vision/visual field	Best-corrected visual acuity test,* slit lamp biomicroscopy,* visual field (automated static or kinetic)*
Elevated intraocular pressure	Applanation tonometry*
Retinal pigmentary degeneration	Fundoscopy,* retinoscopy/refractometry,* visual field,* optical coherence tomography,† electroretinography†
Optic nerve involvement (optic disc swelling, papilloedema, optic atrophy)	Fundoscopy,* visual field,* optical coherence tomography,† visual-evoked potential†

*Musculoskeletal system*	
Short neck and short limbs	Clinical examination (including auxological evaluation)*
Short stature	Clinical examination (including auxological evaluation)*
Arthropathy, joint stiffness and contractures	6-minute walk test,* joint range of motion (shoulders, elbows, wrists, knees, hips and ankles)*
Abnormal bone thickness and shape (e.g. malformation of tarsal bones, pelvis, and vertebral bodies)	X-ray (spine, hips and pelvis)*, radiography†
Claw-like hands	Clinical examination*
Spine deformities (kyphosis, scoliosis)	Evaluation of standing and sitting posture and walking ability (6-minute walk test),* cervical spine flexion/extension,* MRI of the cervical spine,† X-ray of the lumbar spine†

*Ear, nose and throat*	
Enlarged, protruding tongue	Clinical examination*
Recurrent ear infections	Medical history (frequency of ear infections),* otological and audiological examinations*
Progressive hearing loss (conductive and sensorineural)	Otological and audiological examinations*
Frequent upper respiratory tract infections	Medical history (frequency of respiratory infections),* vital signs (pulse, respiratory rate, blood pressure, and oxygen saturation in air)* spirometry to measure FVC*
Thick nasal and tracheal secretions	Examination of upper airway*

*Airway*	
Progressive airway obstruction, tracheobronchomalacia	Examination of upper airway for hypertrophy of the tonsils and adenoids, and tracheal deformities*
Sleep apnoea	Sleep study (assessment of thoracic and abdominal motion; pulse oximetry to measure arterial oxygen saturation and pulse rate; electrocardiography)*

*Skin*	
Thickened and inelastic skin	Clinical examination*
Pebbly, ivory-coloured skin lesions	Clinical examination*

*Abdomen/gastrointestinal system*	
Hepatomegaly	Clinical examination,* abdominal ultrasound,* abdominal MRI†
Splenomegaly	Clinical examination,* abdominal ultrasound,* abdominal MRI†
Bladder obstruction	Abdominal ultrasound*
Chronic diarrhoea	Medical history*
Umbilical and/or inguinal hernias	Clinical examination*

*Psychological wellbeing*	
Poor quality of life	Patient interview,* patient-completed quality of life questionnaires (e.g. Child Health Assessment Questionnaire Disability Index Score [CHAQ DIS], Short Form 36 Health Survey [SF-36])†

Characteristic features of mucopolysaccharidosis type II and the methods available to assist diagnosis [2,3,11,12,46,47,57,75,80,81,84,88,89,97,98,108-129].

All assessments are subject to patient cooperation; *Essential assessment. †Optional assessment.

CT = computed tomography. FVC = forced vital capacity. MRI = magnetic resonance imaging.

## Disease-specific approaches to treating MPS II

Once a diagnosis of MPS II has been confirmed, the available treatment options should be discussed with the patient and his or her parents. An explanation of potential treatment outcomes and adverse events should be given, and realistic treatment goals should be set (CO). Enzyme replacement therapy (ERT) with recombinant I2S (idursulfase, Elaprase^®^; Shire Human Genetic Therapies, Inc., Cambridge, MA, USA) is commonly used to treat MPS II [3]. Alternatives such as stem cell transplantation (STC) using umbilical cord blood, peripheral blood haematopoietic cells or bone marrow have also been used, but they appear to offer limited clinical benefits in patients with this disease and have been associated with a serious risk of morbidity and mortality [31,32]. In many centres, STC is no longer proposed as a therapy for MPS II, although it must be stated that the literature is biased by the reporting of very poor outcomes in patients treated after the onset of significant cognitive decline (CO). Very few boys have received SCT in the early weeks or months of life (see below).

### Enzyme replacement therapy

Idursulfase is a purified form of I2S, produced by recombinant DNA technology in a continuous human cell line. Intravenous ERT with idursulfase provides exogenous enzyme for selective uptake into cells via mannose-6-phosphate receptors on the cell surface [33]. Upon internalisation, the enzyme is transferred and localised within lysosomes, where it catabolises accumulated GAGs [3].

**Figure 3:**
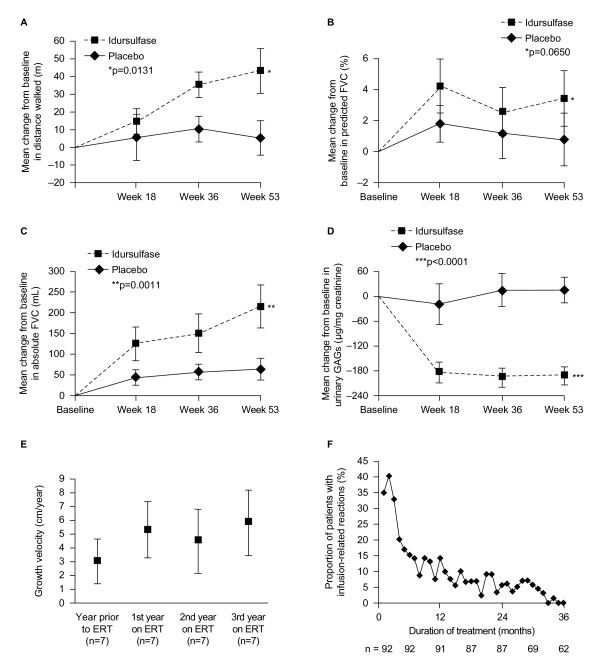
**Clinical effects of enzyme replacement therapy (ERT) with idursulfase (0.5 mg/kg weekly) or placebo in patients with mucopolysaccharidosis type II (MPS II)**. (**a) **Mean (± SE) change from baseline in distance walked in 6-minute walk test; **(b) **mean (± SE) change from baseline in percentage of predicted forced vital capacity; **(c) **mean (± SE) change from baseline in absolute forced vital capacity; **(d) **mean (± SE) change from baseline in concentration of urine glycosaminoglycans; **(e) **mean (± SD) growth velocity before and during enzyme replacement therapy; **(f) **incidence of infusion reactions during treatment with idursulfase. Absolute forced vital capacity is a better measure of respiratory function than percentage of predicted forced vital capacity, as the latter assumes both normal growth and height, which does not apply to patients with MPS II. **(a-d) **adapted from [34] with permission; **(e) **reproduced from [37] with kind permission from Springer Science & Business Media; **(f) **reproduced from [35] with permission. *p = 0.0131; **p = 0.011; ***p < 0.0001, compared with placebo based on analysis of covariance. SD = standard deviation. SE = standard error. ANCOVA = analysis of covariance. FVC = forced vital capacity. ERT = enzyme replacement therapy.

Idursulfase is indicated for the long-term treatment of patients with MPS II. In a randomised, placebo-controlled clinical trial, intravenous administration of idursulfase (0.5 mg/kg body weight weekly for up to 53 weeks) to 32 patients was associated with significant improvements in a composite endpoint comprising change in distance walked in 6 minutes and percentage of predicted forced vital capacity (%FVC) compared with patients receiving placebo (p = 0.0049) [34]. When evaluated individually after 53 weeks, the increase from baseline in mean (± SEM) distance walked in the 6-minute walk test was significantly greater in patients receiving idursulfase compared with those given placebo (+44.3 ± 12.3 m versus +7.3 ± 9.5 m, respectively; p = 0.0131; Figure 3a). %FVC increased more from baseline in patients treated with idursulfase than in the placebo group, although this difference did not reach significance (p = 0.0650; Figure 3b) [34]. The mean increase in absolute FVC from baseline was significantly greater in patients treated weekly with idursulfase compared with placebo (+0.22 ± 0.05 L versus +0.06 ± 0.03 L; p = 0.0011; Figure 3c). The mean decrease in liver volume at 53 weeks was significantly greater in patients treated with idursulfase compared with placebo (-25.3 ± 1.6% versus -0.8 ± 1.6%, respectively; p < 0.0001) as was the change in spleen volume (-25.1 ± 2.4% versus +7.2 ± 4.2%, respectively; p < 0.0001) [34]. Urinary GAG levels were significantly reduced from baseline in patients receiving ERT compared with placebo (mean change, -189.2 ± 25.8 μg/mg creatinine vs. +18.2 ± 29.9 μg/mg creatinine, respectively; p < 0.0001; Figure 3d) [34]. Aside from an improvement in elbow mobility between the weekly idursulfase group compared with placebo (p = 0.0476), no other significant differences between treatment groups for any joint range of motion were found [34].

Following this trial, a 2-year open-label extension study of weekly idursulfase was undertaken in a population of all 94 patients that completed the Phase II/III study [35]. Although %FVC did not change significantly except at a single timepoint, there was a sustained improvement in mean absolute FVC compared with the initial study's baseline (mean change at 3-year timepoint, +0.31 ± 0.06 L, 25.1%; p < 0.05) [34,35]. Increases in distance walked in 6 minutes compared with baseline were maintained, although variable from one assessment to another. The largest increase was seen at 20 months after the start of the initial study - a mean increase of 42 ± 10 m from baseline (p < 0.01) [35]. Effects on liver and spleen volume were also maintained, and a sustained reduction in urinary GAG levels was observed during 3 years of treatment, with a final mean value of 81.7 μg/mg creatinine (well below the upper limit of normal) [35]. Joint range of abduction and flexion-extension improved for the shoulder to a degree felt to be clinically important (approximately 12° and 15°, respectively, at 36 months; both p ≤ 0.005) and remained stable in the elbow, wrist, digits, hip, knee and ankle [35]. After 24 months, both parent- and child-assessed measures of quality of life showed significant improvements from baseline (change in parent-assessed Disability Index Score, -0.13 ± 0.06, p = 0.047; change in child-assessed Disability Index Score, -0.15 ± 0.65, p = 0.031) [35].

Similar findings have been reported in a separate 12-month, open-label, clinical study of 10 adult Japanese patients with attenuated forms of MPS II [36]. Idursulfase has also been found to have a positive influence on growth in 18 patients with MPS II, especially in children younger than 10 years of age (Figure 3e) [37]. In this group a mean height increase of 14.6 cm was seen over 3 years of ERT.

#### Adverse events associated with ERT

As with any intravenous protein product, anaphylactoid reactions, which can be life-threatening in extreme cases, have been observed in some patients treated with idursulfase [33-35,38]. These hypersensitivity responses are more commonly known as infusion-related reactions (IRRs). Late emergent signs and symptoms of IRRs have been observed as long as 24 hours after an initial reaction [38], although these are often mild and do not require hospitalisation. Patients who experience moderate-to-severe IRRs should be monitored for at least 24 hours.

In clinical trials, most of the adverse events reported were related to the underlying disease rather than ERT [33-36]. The most common treatment-related adverse events were IRRs (e.g. headache, hypertension, erythema, pyrexia, flushing, pruritus, urticaria, and/or rash) [33-36]. In general, rates of IRRs tended to decline over time (Figure 3f) and no patient discontinued treatment due to an IRR during clinical studies [34,35]. Immunoglobulin (Ig) G antibodies occurred in 46.9% of patients treated with idursulfase, and IgM antibodies, of unknown importance, were reported in one patient [34]. In approximately half of antibody-positive patients, antibody titres fell below the level of detection after continued treatment [35]. Neutralising antibodies were detected in 22 out of 94 patients. Changes in distance walked in 6 minutes, liver and spleen volume and urinary GAG levels did not appear to be affected by neutralising antibody status [35]. However, individuals with neutralising antibodies showed smaller increases in absolute FVC compared with patients without neutralising antibodies [35]. Further studies into the long-term impact of neutralising antibodies on clinical response are needed.

Serious adverse events were reported in a minority of patients in clinical trials [33-35]. In the randomised placebo-controlled clinical trial, two deaths occurred [34]. Both were associated with pulmonary infections and one of the patients died following a cardiac arrest. Neither death was considered to be related to the study medication. In the subsequent 2-year extension study, one further death occurred owing to upper airway obstruction [35]. Again, this was not considered to be related to the study drug. Hypoxic episodes during enzyme infusion, which necessitate oxygen therapy, have been reported in patients with severe underlying obstructive airway disease (sometimes with pre-existing tracheostomy). In one patient with a febrile respiratory illness, idursulfase administration was associated with hypoxia during the infusion, resulting in a short seizure [33]. These events did not recur with subsequent administration using a slower infusion rate and premedication with low-dose corticosteroids, antihistamines and beta-agonist nebulisation.

#### Administration of ERT

Idursulfase should be administered weekly at a dose of 0.5 mg/kg body weight by intravenous infusion over 3 hours. Although the drug's prescribing information states that the infusion period may be gradually reduced to 1 hour if no IRRs are observed, shortening the infusion time substantially is undesirable, as it may increase the risk of IRRs (CO). Infusion should be supervised by a physician or other healthcare professional experienced in the management of patients with MPS II or other inherited metabolic disorders. Delivery methods include use of a venous cannula or of a totally implantable venous access device, which is placed under general anaesthetic. The patient should be observed closely throughout the infusion and vital signs should be monitored regularly. Caution is required for patients with a febrile infection, airway abnormalities, respiratory distress, or a history of allergies. Reduction in infusion rate and premedication with antihistamines, antipyretics and/or low-dose corticosteroids may be used to reduce the risk of IRRs. Premedication should be given no more than 30 minutes before starting the infusion (CO).

**Figure 4:**
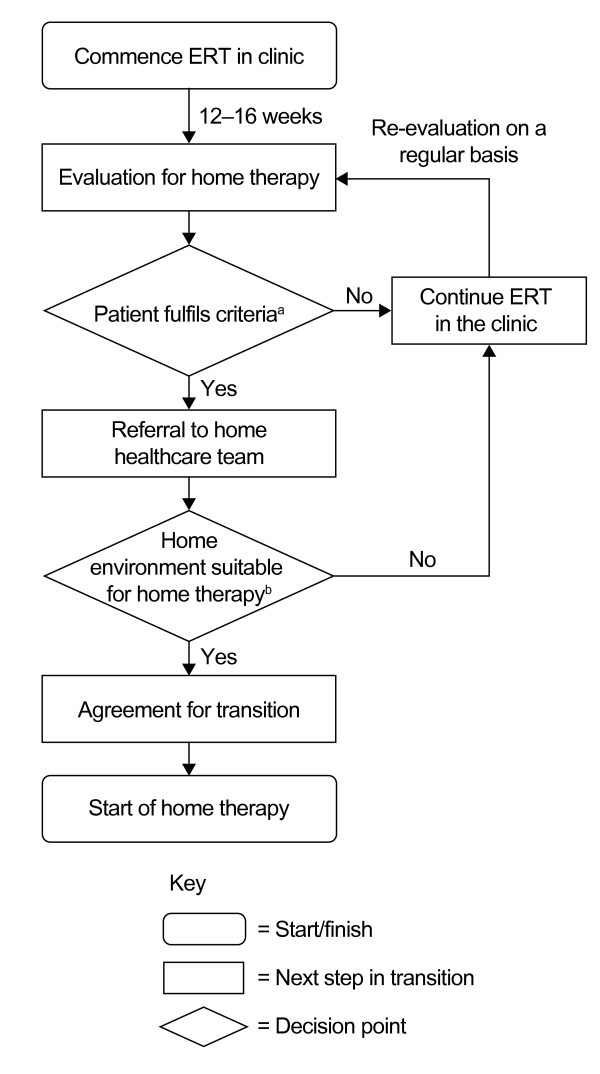
**Algorithm for the provision of enzyme replacement therapy (ERT) outside of the hospital setting**. *^a^*Patient aged 5 years or older, with no infusion-related reactions, with stable airway disease and established intravenous access. *^b^*Under some circumstances, an environment other than the home, such as school, may be considered as an alternative to the clinic. Adapted from [42] with permission from Elsevier.

Following a change to the European product license for idursulfase in March 2010, infusion of ERT at home can now be considered for some patients. Although 'homecare' is not provided in all countries in Europe, it has been associated with increased patient adherence compared with receiving infusions in hospital, and patients and families often report improved quality of life [39-41]. Safety is the primary consideration when providing ERT in the home setting, and strict protocols have been developed to manage the transition from hospital to home care (Figure 4) [42]. Prior to initiation of homecare, the patient's home must be assessed to ensure that it is safe for both the child and the nurse delivering the infusion [43,44]. Patients must have received ERT in hospital for 3-6 months; if patients have previously had IRRs, they must be under control with premedication, and they must not have had an IRR in the 2-8 weeks before homecare is approved and premedication must be given [44]. If a patient has significant respiratory disease (%FVC, 40% or less; or evidence of serious obstructive airway disease), homecare may not be suitable [44].

#### Considerations for initiating and ending treatment

Many patients with MPS II will develop potentially life-threatening manifestations by the second decade of life, so timely treatment is important. The relationship between progressive GAG storage and clinical manifestations in MPS II provides a strong argument for the initiation of ERT as early as possible following diagnosis (CO). Clinical trials of idursulfase demonstrated clinical benefits of treatment in patients older than 5 years [33,34,36], but experience of ERT in younger children is growing [45]. Some national guidelines recommend that ERT should be initiated for all patients with a biochemically confirmed diagnosis of MPS II, including those younger than 5 years [46].

Idursulfase does not cross the blood-brain barrier. Thus, it will not affect the cognitive and behavioural manifestations of MPS II. Weekly intravenous therapy can place a significant burden on both the patient and their family [3,47]; nonetheless, even for patients with advanced disease, ERT may significantly improve quality of life through improvements in respiratory function, organomegaly, and joint mobility [3,47]. In some countries it is recommended that for patients older than 5 years who already have evidence of considerable cognitive decline, the decision to initiate treatment should be at the clinician's discretion, in discussion with the child's parents [46]. Owing to the heterogeneous nature of MPS II and the variable rate of progression, it would seem reasonable to offer ERT to all patients for a 'trial' period of at least 12-18 months, regardless of phenotype, after which time a decision should be made, in consultation with the parents, as to whether to continue (CO). Short- and long-term effects of treatment continuation or cessation on the patient's quality of life should be the primary concern. Thus, any evidence of central nervous system (CNS) disease progression should be taken into consideration when formulating a management strategy (CO).

ERT is not indicated for patients who are pregnant or lactating, or in individuals whose disease is so far advanced that there is little prospect of ERT having any benefit [46]. There are no published data concerning any effect of idursulfase on spermatogenesis; therefore, men with MPS II should continue to receive ERT when trying to conceive (CO). Treatment is also not recommended if the patient has a comorbid life-threatening disease for which the prognosis is unlikely to be influenced by ERT [46].

Treatment should be discontinued for patients with life-threatening IRRs that are not adequately prevented or controlled by antihistamines and corticosteroids (CO). In these circumstances, the possibility of potentially fatal sudden respiratory failure is high.

### Stem cell transplantation

Transplantation of stem cells using bone marrow, peripheral blood haematopoietic cells or umbilical cord blood has been shown to be effective in slowing disease progression in selected lysosomal and peroxisomal inherited metabolic storage diseases, including MPS IH (Hurler syndrome), MPS VI (Maroteaux-Lamy syndrome), X-linked adrenoleukodystrophy, metachromatic leukodystrophy and globoid-cell leukodystrophy (Krabbe disease) [48-54]. SCT relies on the progressive replacement throughout the body of endogenous haematopoietic lineage cells with exogenous cells transplanted from a healthy donor. Importantly, experiments in mice have shown that the transplanted cells can migrate across the blood-brain barrier, differentiate into microglia, and express lysosomal enzymes that can be taken up by cells in the CNS and delivered to the lysosome [55]. This, coupled with the apparent inability of enzymes administered intravenously to cross the blood-brain barrier, has stimulated interest in the therapeutic potential of STC for preventing or treating the neurological manifestations of metabolic storage diseases, including MPS II.

At the time of writing, no controlled clinical studies have been conducted to evaluate the effects of bone marrow transplantation (BMT), haematopoietic stem cell transplantation (HSCT) or umbilical cord blood transplantation (UCBT) in patients with MPS II; with experience limited to single published case studies or small case series. This is hardly surprising given the rarity of MPS II, and it is unlikely that formal randomised controlled trials will ever be conducted.

The use of STC for the treatment of MPS II is controversial because of the profound risk of morbidity and mortality associated with this treatment approach [31]. Many patients receive immunosuppressant and steroid medication following transplantation to protect against or alleviate graft-versus-host disease [56]. However, the use of immunosuppressants can leave the patient vulnerable to infection, and the chronic use of steroids may lead to orthopaedic complications (e.g. osteonecrosis of the hip) [47].

#### Bone marrow transplantation

The majority of clinical experience of BMT in patients with MPS II comes from observations made in case studies. At the time of writing, only two studies have examined the long-term outcomes of BMT in groups of four or more patients with MPS II [31,32]. In these studies, BMT was found to increase or normalise I2S activity in leukocytes, but not serum, and was associated with decreased or normalised urinary GAG excretion in patients with MPS II [31,32]. The long-term outcomes of BMT appear unpredictable in patients with MPS II, perhaps owing to the heterogeneity of the disease. Resolution of hepatosplenomegaly, improvement of upper airway obstruction, progressive resolution of coarsened facial features, reduced joint stiffness, stabilisation of perceptual hearing defects and improvement in transmission hearing defects were all reported [32]. Cardiac structure and function were found to stabilise in some patients [32]; an important finding given that cardiac failure is a common cause of death in individuals with MPS II [57]. However, no quantitative data are available for any of these observations.

BMT does not appear to improve neurological function in patients with a severe phenotype if they already have signs of developmental delay or neurological involvement at transplantation [31,32]. By contrast, stabilisation of neurological function following BMT has been reported in patients with MPS II with an attenuated phenotype who were followed for 7-17 years [32]. Whether this observation can be attributed to BMT is unclear.

Observations from individual case studies include stabilisation of disease, resolution of hepatosplenomegaly and skin tightness, and improved joint mobility and growth, each contributing to increased quality of life [58-61]. Clearance of GAGs from hepatocytes has also been described [62]. Reports on the effect of BMT on the progression of neurological manifestations are inconsistent, with no deterioration in some patients [59], but clear deterioration in others despite transplantation at a young age [63].

#### Haematopoietic stem cell transplantation

As with BMT, there are very few data on the effects on HSCT in patients with MPS II. Notable observations include a reduction in levels of a urinary marker of GAG levels (measured as dermatan sulphate to chondroitin sulphate ratio) in one patient [64], and complete resolution of skin papules and a progressive reduction in skin tightness in five boys (aged 4-11 years) within 35 days of HSCT [65].

#### Umbilical cord blood transplantation

Improvement or complete resolution of hepatomegaly has been reported in patients with mild and severe forms for MPS II following UCBT [56,66]. Urinary GAG excretion was normalised in a patient with an attenuated form of MPS II, and growth and development remained normal up to 2 years after transplantation [56]. No other physiological changes following UCBT have been reported. Detection of very low I2S levels in the brain of a patient with a severe form of MPS II suggests that stem cells delivered by UCBT may be able to penetrate the CNS in individuals with MPS II, although enzyme levels were insufficient for metabolic improvement [66].

Overall, it appears that SCT can have some beneficial effects on the peripheral signs and symptoms of MPS II. However, it is clear that this approach does not preserve or improve neurological function in patients with severe forms of MPS II if they already show signs of neurological deterioration. The long-term effects of BMT, HSCT and UCBT on neurological function in patients with more attenuated forms of MPS II require further study. In the absence of a clear long-term effect on the neurological manifestations of MPS II, the potential clinical benefits of successful engraftment do not appear to outweigh the immediate and medium-long-term risks of the procedure, particularly when other effective and well-tolerated treatments for peripheral manifestations are available (CO).

### Therapies in development

Owing to the dearth of therapeutic options for alleviating the neurological manifestations of LSDs, much research has focused on the development of well-tolerated therapies that can cross the blood-brain barrier. One approach that has been explored is the infusion of ERT into the cerebrospinal fluid, thereby enabling widespread distribution throughout the CNS. Experiments in animal models have yielded promising results [67], and a study into the feasibility and safety of intrathecally delivered idursulfase in patients with MPS II is underway.

Other areas of research include the use of pharmacological chaperones, gene therapy and substrate reduction therapy. Although a comprehensive overview of these therapeutic options is beyond the scope of this article, all have been found to cross the blood-brain barrier and promising findings have been reported *in vitro *and in animal models [68-72]. It is hoped that in the near future these therapies may help to prevent or reverse the neurological manifestations observed in patients with severe forms of MPS II.

## Non-disease-specific approaches to managing MPS II

The wide range of effects of MPS II on the body and the severity of many of the manifestations mean that most patients will require substantial medical and surgical support, even if they are receiving ERT or have received SCT. A full review of this broad topic is not possible here, so key aspects of multidisciplinary care are presented.

### Management of cardiovascular manifestations

Cardiovascular manifestations develop at a young age in patients with MPS II, and most patients exhibit at least one cardiovascular sign or symptom by the second decade of life [3]. Typical changes include valve disease (e.g. changes in morphology and impaired function: affecting mitral, aortic, tricuspid and pulmonary valves in decreasing frequency), ventricular hypertrophy, hypertension, and arrhythmia (e.g. tachycardia, bradycardia, atrioventricular block) [3,14,73]. The progression of cardiac involvement must be monitored closely, and patients should undergo regular echocardiography, electrocardiography, and Holter monitoring, if indicated (Table 1).

Valve disease affects more than half of patients and can lead to ventricular hypertrophy or heart failure [3,12]. In some countries, prophylactic antibiotic therapy may be given before any surgical or major dental procedure as a precaution. However, this practice is no longer recommended by the National Institute for Health and Clinical Excellence [74]. Valve replacement has been reported, but remains uncommon [75,76]. Hypertension is typically under-diagnosed in patients with MPS II and should be treated using standard agents, such as angiotensin-converting enzyme inhibitors, angiotensin receptor blockers, diuretics and calcium-channel blockers (CO). Arrhythmias should be treated with ablation, antiarrhythmic drugs, anticoagulants and, if necessary, placement of an implantable cardioverter defibrillator (CO).

### Management of neurological involvement

Depending on disease severity, neurological manifestations of MPS II may include a delay in achieving developmental milestones, cognitive impairment and seizures [11,12]. In addition, communicating hydrocephalus, spinal cord compression, and carpal tunnel syndrome (CTS) typically require surgical intervention [47]. Impaired cognition or delayed speech in children of preschool age should provoke further investigation with cognitive tests, and behavioural therapy and/or the use of behaviour-modifying medication may be necessary (CO). Seizures can usually be controlled by anticonvulsant therapy [47]. To avoid unwanted adverse events, low-dose monotherapy is preferred (CO). For patients with communicating hydrocephalus or evidence of progressive ventricular enlargement, a ventriculoperitoneal shunt can be placed to relieve intracranial pressure [77,78]. Motor function has been reported to improve after shunt placement [79].

A common feature of MPS II is progressive compression of the spinal cord with resulting cervical myelopathy [47,80]. This can lead to reduced activity, difficulty in rising from a sitting position, paresis and spasticity, pain or loss of sensation in the upper and lower body, as well as bladder and bowel dysfunction. Irreversible cord damage can occur if this is left untreated, so surgical decompression should be considered as soon as symptoms occur [81-83]. Similarly, decompression of the median nerve is recommended for patients with demonstrated loss of hand sensation and/or function or abnormal nerve conduction studies (CTS). Both decompression procedures have been associated with rapid and sustained improvement in sensation and function, as well as providing pain relief [47,84-87].

### Management of ocular manifestations

Ocular involvement in MPS II generally consists of loss of vision, optic disc swelling, papilloedema, optic atrophy and retinal pigmentary degeneration [2,88]. Corneal clouding is almost never encountered [2]. Treatment of ocular complications in patients with MPS II does not differ substantially from approaches used for otherwise healthy individuals (see Table 1). If glaucoma occurs, most patients respond well to intraocular pressure-lowering eye drops (CO). If optic nerve involvement occurs due to raised intracranial pressure, this should be addressed using standard methods. Unfortunately, optic involvement associated with GAG accumulation or retinal degradation cannot be treated, although patients do benefit from magnifying devices when reading (CO).

### Management of musculoskeletal manifestations

Typical musculoskeletal manifestations of MPS II include short stature, spine deformities, joint stiffness, contractures, and claw-like hands [89]. Orthopaedic therapy should be considered for patients with musculoskeletal manifestations, as this can help to address psychosocial aspects of the disease, such as loss of mobility and independence, as well as relieving the symptoms themselves. Non-surgical approaches include physiotherapy and the use of orthopaedic devices, such as orthotic footwear, braces, corsets and walking aids, to assist with daily living activities. These approaches can also help maximise muscle strength and range of movement [89]. Surgical procedures include decompression of the spinal cord or median nerve, instrumented fusion (to stabilise and strengthen the spine), arthroscopy, hip or knee replacement, and correction of the lower limb axis [89,90].

It has been suggested that recombinant human growth hormone (GH) may help overcome short stature in patients with MPS II [91]. GH therapy has been shown to be well tolerated and effective in improving linear growth in patients with GH deficiency, Turner syndrome and other growth disorders [92-94]; however, experience in patients with MPS II is very limited. The only published report of GH therapy in patients with MPS II is provided by Polgreen & Miller, who observed transient increases in growth velocity in two patients treated for up to 1 year [91]. Although GH therapy was well tolerated in these patients, there are currently insufficient data on the safety and efficacy of this approach in children with MPS II to recommend it as a standard of care (CO). Furthermore, rapid growth carries the theoretical risk of worsening of orthopaedic complications typically observed in patients with MPS II [95]. With this in mind, patients with MPS II who are prescribed GH therapy must be followed closely by orthopaedic physicians who are familiar with MPS diseases [96].

### Management of ear, nose and throat manifestations

Ear, nose and throat features of MPS II include hearing loss, recurrent otitis media, an enlarged tongue, hypertrophic adenoids and tonsils and progressive airway obstruction [97,98]. Chronic and recurrent (more than six episodes per year) upper-respiratory tract infections are common, and affected patients may benefit from analysis of functional antibodies to S*treptococcus pneumoniae *and *Haemophilus influenzae*, with booster vaccinations being provided when appropriate (CO). In the absence of a correctable immune deficiency, adenotonsillectomy or ventilation tube insertion may be appropriate for patients with severe signs and symptoms (CO). If hearing loss occurs secondary to persistent middle ear effusion, the possibility of providing hearing aids or inserting ventilation tubes should be discussed with the patient and their parents. Both treatments are effective, but hearing aids are preferred for children with significant comorbidity (CO). Macroglossia secondary to GAG storage is very difficult to manage. Operations on the tongue are not indicated in patients with MPS II, as the risk of postoperative respiratory obstructions is high (CO).

### Management of airway abnormalities

Upper airway obstruction is a major contributor to the premature mortality seen in MPS II [13]. It is thought to result from progressive deposition of GAGs in the soft tissues of the throat and trachea, and may lead to obstructive sleep apnoea [98]. Initial treatments for obstructive sleep apnoea include nocturnal supplemental oxygen. Tonsillectomy and adenoidectomy may be performed when these are enlarged [90,97], but, because of the progressive involvement of the throat and trachea, improvements may only be partial and/or temporary. Continuous positive airway pressure (CPAP) can be used to splint the airway open during sleep, and has been associated with marked improvements in sleep quality [98,99], leading to reduced fatigue during the following day and fewer complaints of headache [98]. For patients for whom CPAP is not well tolerated, tracheostomy may be used to bypass the upper airway obstruction or to support the trachea when there is significant collapse due to tracheobronchomalacia. However, complications following this procedure are common and can be life-threatening [100], so caution is advised (CO).

Patients with MPS II should undergo regular examination of the upper airway for signs and symptoms of developing airway obstruction, and an overnight sleep study should be conducted in patients with obstructive sleep apnoea (Table 1) [47,98]. For a more thorough evaluation of the airway, bronchoscopy may be performed [47]. Routine monitoring of pulmonary function is challenging, as spirometry requires the full cooperation of the patient and is effort dependent. It cannot be used reliably for children younger than 6-7 years of age and may be impossible for patients with significant CNS involvement [98].

### Surgical intervention

Surgical intervention is often required at a young age to address the clinical manifestations of MPS II [75,76,90]. The most common procedures are insertion of ventilation tubes, hernia repair, adenoidectomy, tonsillectomy, and median nerve decompression [90]. Surgery may sometimes precede diagnosis, so it is important to evaluate a patient's surgical history when a diagnosis of MPS II is suspected [90].

### Management of anaesthetic risk

The short neck, immobile jaw, and pathological changes in the upper airways make general anaesthesia for patients with MPS II a difficult and high-risk procedure [98]. For this reason, it is good practice to consider local or regional anaesthesia where possible. Combining minor surgical procedures may be appropriate in some instances; however, extending the operation time increases the risks of respiratory complications dramatically, so caution is advised (CO).

Before surgery, the patient should be assessed by a multidisciplinary team that includes a cardiologist, otorhinolaryngologist and anaesthetist. A full cardiac assessment is necessary (CO). The severity of obstructive sleep apnoea can be assessed with a sleep study or more formal polysomnography. If possible, flexible nasendoscopy and a computed tomography scan of the airway should be carried out preoperatively to evaluate the anatomy of the airway [101,102]. Tracheomalacia of the airway makes visualisation and subsequent endotracheal intubation problematic (Figure 1e) [103,104]. Extubation presents another major risk, as postobstruction pulmonary oedema may exacerbate upper-airway obstruction and has been reported to occur as late as 27 hours after surgery [97,105,106]. Some patients may be unable to maintain the airway after extubation, resulting in the need for urgent reintubation or tracheostomy. Early extubation can reduce this risk substantially [98]. As a rule, it is recommended that patients with MPS II should only undergo surgery at centres with experience of the perioperative management of individuals with this disease, and on-site intensive care facilities [98,106].

## Social aspects of MPS II

Patients with MPS II and their families generally require considerable psychological and social support following diagnosis and before and after treatment. Clinicians should be prepared to provide guidance on treatment-related issues and to answer questions concerning carrier status and prenatal diagnosis. Genetic counselling should also be offered to family members.

Patient societies provide vital psychosocial support to parents and siblings through one-to-one counselling, as well as providing links to other affected individuals through befriending schemes and regional family days. They play a key role in helping patients and their families to understand their disease, and can make parents aware of disability benefits, respite care, and housing help for which they may be eligible. Both verbal and written information is provided regarding issues such as education, grants, equipment, care plans, independent living, and pre- and post-bereavement support.

In recent years, patient societies have worked closely with physicians, specialist nurses and homecare companies to make new treatment options available to patients. For instance, in the UK, the Society for Mucopolysaccharide Diseases [107] played an important role in enrolling patients into idursulfase clinical trials, and continues to assist patients who want to take part in ongoing investigations by ensuring that patients and their families are adequately supported. Importantly, patient societies also can help gather valuable information on how treatment affects quality of life by encouraging their members to participate in surveys.

## Summary and conclusions

MPS II is a rare, inherited disease that affects multiple organs and systems. The development of ERT with idursulfase has provided a means of addressing the underlying lysosomal enzyme deficiency, and improvements in certain somatic signs and symptoms have been reported in clinical studies. Unfortunately, options for alleviating the neurological manifestations of MPS II remain limited: intravenously administered idursulfase does not pass through the blood-brain barrier, and experience of intrathecal administration of ERT is restricted to small clinical trials. There are few published data on long-term outcomes from STC in MPS II, especially regarding neurological function. Further work is required to ascertain whether the potential benefits of successful engraftment outweigh the risks of the procedures, particularly as an effective treatment of certain peripheral manifestations is already available. Given the heterogeneous presentation of this disorder, a wide range of specialties is likely to be involved in its diagnosis and most patients require substantial medical, surgical, and psychosocial support. Thus, close collaboration between all involved in dealing with MPS II is essential if patients are to be diagnosed as early as possible and treated safely and effectively.

## Abbreviations

BMT: bone marrow transplantation; CNS: central nervous system; CO: consensus opinion; CPAP: continuous positive airway pressure; CTS: carpal tunnel syndrome; DNA: deoxyribonucleic acid; ERT: enzyme replacement therapy; FVC: forced vital capacity; %FVC: percentage of predicted forced vital capacity; GAGs: glycosaminoglycans; GH: growth hormone; HSCT: haematopoietic stem cell transplantation; HSEEC: Hunter Syndrome European Expert Council; I2S: iduronate-2-sulfatase; *IDS*: iduronate-2-sulfatase gene; *IDS2*: iduronate-2-sulfatase pseudogene; Ig: immunoglobin; IRRs: infusion-related reactions; LSDs: lysosomal storage diseases; MPS IH: mucopolysaccharidosis type IH (Hurler syndrome); MPS II: mucopolysaccharidosis type II (Hunter syndrome); MPS VI: mucopolysaccharidosis type VI (Maroteaux-Lamy syndrome); STC: stem cell transplantation; UCBT: umbilical cord blood transplantation.

## Competing interests

MS has received honoraria and travel expenses for presenting at scientific meetings and sitting on advisory boards sponsored by BioMarin Pharmaceutical, Inc., Genzyme Corporation and Shire Human Genetic Therapies, and is a member of the HSEEC, which receives financial support from Shire Human Genetic Therapies. MS also receives honoraria for development of educational presentations for Genzyme Corporation and Shire Human Genetic Therapies. MS's institution receives research grants from Genzyme Corporation and Shire Human Genetic Therapies.

ZA has received reimbursement for presenting lectures at symposia arranged by Shire Human Genetic Therapies and partial funding for travel to these symposia. ZA is a member of the HSEEC, which receives financial support from Shire Human Genetic Therapies.

MB has received honoraria, travel support and unrestricted grants from Shire Human Genetic Therapies, Genzyme Corporation, BioMarin Pharmaceutical, Inc. and Actelion Pharmaceuticals Ltd. MB is a member of the HSEEC, which receives financial support from Shire Human Genetic Therapies.

OB is a member of a Speakers' Bureau for Shire, Inc. OB is a member of the HSEEC, which receives financial support from Shire Human Genetic Therapies.

IAB has received travel grants and honoraria from Shire Human Genetic Therapies to attend and deliver lectures at medical meetings.

LDM has received honoraria and travel expenses for presenting at scientific meetings and sitting on advisory boards sponsored by Genzyme Corporation and Shire Human Genetic Therapies. LDM is a member of the HSEEC, which receives financial support from Shire Human Genetic Therapies.

NG's institution has received funding from Shire Human Genetic Therapies for involvement in a patient registry, clinical trials and for travel and accommodation during a LSD symposium. NG is a member of the HSEEC, which receives financial support from Shire Human Genetic Therapies.

EG-N has received travel grants from Shire Human Genetic Therapies. EG-N is a member of the HSEEC, which receives financial support from Shire Human Genetic Therapies.

PH has no competing interests.

SJ has received travel assistance and honoraria for lectures, and consultancy fees from Shire Human Genetic Therapies. SJ is a member of the HSEEC, which receives financial support from Shire Human Genetic Therapies.

WK has received honoraria for giving lectures at symposia arranged by Shire Human Genetic Therapies and partial funding for travel to the symposia.

CK has received research funding, consultancy fees and/or speaker's fees from Shire Human Genetic Therapies, Genzyme Corporation, Actelion Pharmaceuticals Ltd and BioMarin Pharmaceutical, Inc.

CL has received speaker's honoraria from Shire Human Genetic Therapies, BioMarin Pharmaceutical, Inc. and Genzyme Corporation and partial funding for medical advice.

CAL has received reimbursement of expenses and honoraria from Shire Human Genetic Therapies and Genzyme Corporation. The Society for Mucopolysaccharide Diseases (UK) has received educational grants from Genzyme Corporation, Shire Human Genetic Therapies, BioMarin Pharmaceutical, Inc. and Amicus Therapeutics, Inc. The Society for Mucopolysaccharide Diseases also receives fees and reimbursement of expenses in respect of clinical trial patient access from BioMarin Pharmaceutical, Inc. and Shire Human Genetic Therapies.

ELT has received funding for travel and expenses for attendance at scientific meetings sponsored by Shire Human Genetic Therapies and is a member of the HSEEC, which receives financial support from Shire Human Genetic Therapies.

BL has received honoraria and travel expenses for attending and presenting at scientific meetings and sitting advisory boards sponsored by Shire Human Genetic Therapies, Genzyme Corporation and BioMarin Pharmaceutical, Inc. BL has also received honoraria for providing writing, medical and/or administrative support to Shire Human Genetic Therapies, Genzyme Corporation and BioMarin Pharmaceutical, Inc. BL is a member of the publication steering committee for LSDs, which is sponsored by Shire Human Genetic Therapies. BL also receives honoraria for development of educational presentations for Genzyme Corporation. BL's institution receives research grants from Shire Human Genetic Therapies and BioMarin Pharmaceutical, Inc.

AML has served on scientific advisory boards and as a consultant for Shire Human Genetic Therapies, Zymenex A/S, and Genzyme Corporation. AML has received funding for travel from Genzyme Corporation and Shire Human Genetic Therapies, and receives research support from Shire Human Genetic Therapies and Genzyme Corporation. AML is a member of the HSEEC, which receives financial support from Shire Human Genetic Therapies.

GM has received reimbursements for giving lectures at symposia arranged by Shire Human Genetic Therapies and partial funding for travel to the symposia. GM is a member of the HSEEC, which receives financial support from Shire Human Genetic Therapies.

SP has received travel grants, speakers' honoraria and scientific grants from Shire Human Genetic Therapies, as well as travel grants from BioMarin Pharmaceutical, Inc. and Genzyme Corporation.

MR has no competing interests.

CS has received funding from Shire Human Genetic Therapies for travel to LSD symposia.

AT-S has received speaker's honoraria and is a principal investigator in clinical trials conducted by Shire Human Genetic Therapies. AT-S is a member of the HSEEC, which receives financial support from Shire Human Genetic Therapies.

AvdP is a member of the HSEEC and the Hunter Outcome Survey Board, which receive financial support from Shire Human Genetic Therapies.

RW has no competing interests.

JZ is a member of the HSEEC, which receives financial support from Shire Human Genetic Therapies.

JEW is member of the HSEEC and Global advisory board, which receive financial support from Shire Human Genetic Therapies. JEW has received honoraria and travel expenses for presenting at scientific meetings sponsored by Shire Human Genetic Therapies.

## Authors' contributions

JEW drafted the manuscript, and other authors contributed to the writing. All authors have seen and approved the final manuscript.

## Guarantor

JEW accepts full responsibility for the manuscript and controlled the decision to publish.

## References

[B1] Bach G Eisenberg F Jr Cantz M Neufeld EF The defect in the Hunter syndrome: deficiency of sulfoiduronate sulfatase Proc Natl Acad Sci USA 1973 70 7 2134 2138 10.1073/pnas.70.7.2134 4269173 PMC433682

[B2] Neufeld EF Muenzer J Scriver CR, Beaudet AL, Sly WS, Valle D The mucopolysaccharidoses The metabolic and molecular bases of inherited disease 2001 New York: McGraw-Hill 3421 3452

[B3] Wraith JE Scarpa M Beck M Bodamer OA De Meirleir L Guffon N Meldgaard Lund A Malm G van der Ploeg AT Zeman J Mucopolysaccharidosis type II (Hunter syndrome): a clinical review and recommendations for treatment in the era of enzyme replacement therapy Eur J Pediatr 2008 167 3 267 277 10.1007/s00431-007-0635-4 18038146 PMC2234442

[B4] Baehner F Schmiedeskamp C Krummenauer F Miebach E Bajbouj M Whybra C Kohlschutter A Kampmann C Beck M Cumulative incidence rates of the mucopolysaccharidoses in Germany J Inherit Metab Dis 2005 28 6 1011 1017 10.1007/s10545-005-0112-z 16435194

[B5] Poorthuis BJ Wevers RA Kleijer WJ Groener JE de Jong JG van Weely S Niezen-Koning KE van Diggelen OP The frequency of lysosomal storage diseases in The Netherlands Hum Genet 1999 105 1-2 151 156 10.1007/s004399900075 10480370

[B6] Nelson J Incidence of the mucopolysaccharidoses in Northern Ireland Hum Genet 1997 101 3 355 358 10.1007/s004390050641 9439667

[B7] Tuschl K Gal A Paschke E Kircher S Bodamer OA Mucopolysaccharidosis type II in females: case report and review of literature Pediatr Neurol 2005 32 4 270 272 10.1016/j.pediatrneurol.2004.10.009 15797184

[B8] Sukegawa K Matsuzaki T Fukuda S Masuno M Fukao T Kokuryu M Iwata S Tomatsu S Orii T Kondo N Brother/sister siblings affected with Hunter disease: evidence for skewed × chromosome inactivation Clin Genet 1998 53 2 96 101 9611068 10.1111/j.1399-0004.1998.tb02654.x

[B9] Cudry S Tigaud I Froissart R Bonnet V Maire I Bozon D MPS II in females: molecular basis of two different cases J Med Genet 2000 37 10 E29 10.1136/jmg.37.10.e29 11015461 PMC1757154

[B10] Kloska A Jakóbkiewicz-Banecka J Tylki-Szymanska A Czartoryska B Wergrzyn G Female Hunter syndrome caused by a single mutation and familial XCI skewing: implication for other X-linked disorders Clin Genet 2010 10.1111/j.1399-0004.2010.01574.x 21062272

[B11] Martin R Beck M Eng C Giugliani R Harmatz P Munoz V Muenzer J Recognition and diagnosis of mucopolysaccharidosis II (Hunter syndrome) Pediatrics 2008 121 2 e377 386 10.1542/peds.2007-1350 18245410

[B12] Schwartz IV Ribeiro MG Mota JG Toralles MB Correia P Horovitz D Santos ES Monlleo IL Fett-Conte AC Sobrinho RP A clinical study of 77 patients with mucopolysaccharidosis type II Acta Paediatr 2007 96 Suppl 455 63 70 10.1111/j.1651-2227.2007.00212.x 17391446

[B13] Jones SA Almássy Z Beck M Burt K Clarke JT Giugliani R Hendriksz C Kroepfl T Lavery L Lin SP Mortality and cause of death in mucopolysaccharidosis type II: a historical review based on data from the Hunter Outcome Survey (HOS) J Inherit Metab Dis 2009 32 4 534 543 10.1007/s10545-009-1119-7 19597960

[B14] Wraith JE Beck M Giugliani R Clarke J Martin R Muenzer J Initial report from the Hunter Outcome Survey Genet Med 2008 10 7 508 516 10.1097/GIM.0b013e31817701e6 18580692

[B15] Chamoles NA Blanco MB Gaggioli D Casentini C Hurler-like phenotype: enzymatic diagnosis in dried blood spots on filter paper Clin Chem 2001 47 12 2098 2102 11719472

[B16] Martin R Mucopolysaccharidosis type II Gene Reviews 1993 http://www.ncbi.nlm.nih.gov/bookshelf/br.fcgi?book=gene&part=hunter

[B17] Altarescu G Renbaum P Eldar-Geva T Brooks B Varshaver I Avitzour M Margalioth EJ Levy-Lahad E Elstein D Epsztejn-Litman S Preventing mucopolysaccharidosis type II (Hunter syndrome): PGD and establishing a Hunter (46, XX) stem cell line Prenat Diagn 2011 10.1002/pd.2786 21706504

[B18] Scarpa M Mucopolysaccharidosis type II Gene Reviews 2011 http://www.ncbi.nlm.nih.gov/bookshelf/br.fcgi?book=gene&part=hunter

[B19] Keulemans JL Sinigerska I Garritsen VH Huijmans JG Voznyi YV van Diggelen OP Kleijer WJ Prenatal diagnosis of the Hunter syndrome and the introduction of a new fluorimetric enzyme assay Prenat Diagn 2002 22 11 1016 1021 10.1002/pd.457 12424767

[B20] Archer IM Kingston HM Harper PS Prenatal diagnosis of Hunter syndrome Prenat Diagn 1984 4 3 195 200 10.1002/pd.1970040306 6431402

[B21] Manara R Rampazzo A Cananzi M Salviati L Mardari R Drigo P Tomanin R Gasparotto N Priante E Scarpa M Hunter syndrome in an 11-year-old girl on enzyme replacement therapy with idursulfase: brain magnetic resonance imaging features and evolution J Inherit Metab Dis 2010 10.1007/s10545-009-9023-8 20052546

[B22] Sukegawa K Song XQ Masuno M Fukao T Shimozawa N Fukuda S Isogai K Nishio H Matsuo M Tomatsu S Hunter disease in a girl caused by R468Q mutation in the iduronate-2-sulfatase gene and skewed inactivation of the × chromosome carrying the normal allele Hum Mutat 1997 10 5 361 367 10.1002/(SICI)1098-1004(1997)10:5<361::AID-HUMU5>3.0.CO;2-I 9375851

[B23] Clarke JT Greer WL Strasberg PM Pearce RD Skomorowski MA Ray PN Hunter disease (mucopolysaccharidosis type II) associated with unbalanced inactivation of the × chromosomes in a karyotypically normal girl Am J Hum Genet 1991 49 2 289 297 1678247 PMC1683291

[B24] Mossman J Blunt S Stephens R Jones EE Pembrey M Hunter's disease in a girl: association with X:5 chromosomal translocation disrupting the Hunter gene Arch Dis Child 1983 58 11 911 915 10.1136/adc.58.11.911 6418082 PMC1628393

[B25] Broadhead DM Kirk JM Burt AJ Gupta V Ellis PM Besley GT Full expression of Hunter's disease in a female with an X-chromosome deletion leading to non-random inactivation Clin Genet 1986 30 5 392 398 3100113 10.1111/j.1399-0004.1986.tb01896.x

[B26] Randall DR Colobong KE Hemmelgarn H Sinclair GB Hetty E Thomas A Bodamer OA Volkmar B Fernhoff PM Casey R Heparin cofactor II-thrombin complex: a biomarker of MPS disease Mol Genet Metab 2008 94 4 456 461 10.1016/j.ymgme.2008.05.001 18511319

[B27] Langford-Smith KJ Mercer J Petty J Tylee K Church H Roberts J Moss G Jones S Wynn R Wraith JE Heparin cofactor II-thrombin complex and dermatan sulphate:chondroitin sulphate ratio are biomarkers of short- and long-term treatment effects in mucopolysaccharide diseases J Inherit Metab Dis 2011 34 2 499 508 10.1007/s10545-010-9254-8 21170681 PMC3063559

[B28] Engelbert RHH Custers JWH van der Net J van der Graaf Y Beemer FA Helders PJM Functional outcome in osteogenesis imperfecta: disability profiles using the PEDI Pediatr Phys Ther 1997 9 18 22

[B29] Daltroy LH Liang MH Fossel AH Goldberg MJ The POSNA pediatric musculoskeletal functional health questionnaire: report on reliability, validity, and sensitivity to change. Pediatric Outcomes Instrument Development Group. Pediatric Orthopaedic Society of North America J Pediatr Orthop 1998 18 5 561 571 9746401 10.1097/00004694-199809000-00001

[B30] Beck M Muenzer J Scarpa M Evaluation of disease severity in mucopolysaccharidoses J Pediatr Rehab Med 2010 3 1 39 46 10.3233/PRM-2010-0100 21791828

[B31] Vellodi A Young E Cooper A Lidchi V Winchester B Wraith JE Long-term follow-up following bone marrow transplantation for Hunter disease J Inherit Metab Dis 1999 22 5 638 648 10.1023/A:1005525931994 10399096

[B32] Guffon N Bertrand Y Forest I Fouilhoux A Froissart R Bone marrow transplantation in children with Hunter syndrome: outcome after 7 to 17 years J Pediatr 2009 154 5 733 737 10.1016/j.jpeds.2008.11.041 19167723

[B33] Muenzer J Gucsavas-Calikoglu M McCandless SE Schuetz TJ Kimura A A Phase I/II clinical trial of enzyme replacement therapy in mucopolysaccharidosis II (Hunter syndrome) Mol Genet Metab 2007 90 3 329 337 10.1016/j.ymgme.2006.09.001 17185020

[B34] Muenzer J Wraith JE Beck M Giugliani R Harmatz P Eng CM Vellodi A Martin R Ramaswami U Gucsavas-Calikoglu M A Phase II/III clinical study of enzyme replacement therapy with idursulfase in mucopolysaccharidosis II (Hunter syndrome) Genet Med 2006 8 8 465 473 10.1097/01.gim.0000232477.37660.fb 16912578

[B35] Muenzer J Beck M Eng C Giugliani R Harmatz P Martin R Ramaswami U Vellodi A Wraith JE Cleary M Long-term, open-labeled extension study of idursulfase in the treatment of Hunter syndrome Genet Med 2011 13 2 95 101 10.1097/GIM.0b013e3181fea459 21150784

[B36] Okuyama T Tanaka A Suzuki Y Ida H Tanaka T Cox GF Eto Y Orii T Japan Elaprase Treatment (JET) study: idursulfase enzyme replacement therapy in adult patients with attenuated Hunter syndrome (Mucopolysaccharidosis II, MPS II) Mol Genet Metab 2010 99 1 18 25 10.1016/j.ymgme.2009.08.006 19773189

[B37] Schulze-Frenking G Jones SA Roberts J Beck M Wraith JE Effects of enzyme replacement therapy on growth in patients with mucopolysaccharidosis type II J Inherit Metab Dis 2011 34 1 203 208 10.1007/s10545-010-9215-2 20978944 PMC3026660

[B38] Miebach E Management of infusion-related reactions to enzyme replacement therapy in a cohort of patients with mucopolysaccharidosis disorders Int J Clin Pharmacol Ther 2009 47 Suppl 1 S100 106 20040319 10.5414/cpp47100

[B39] Burton BK Wiesman C Paras A Kim K Katz R Home infusion therapy is safe and enhances compliance in patients with mucopolysaccharidoses Mol Genet Metab 2009 97 3 234 236 10.1016/j.ymgme.2009.04.007 19427803

[B40] Milligan A Hughes D Goodwin S Richfield L Mehta A Intravenous enzyme replacement therapy: better in home or hospital? Br J Nurs 2006 15 6 330 333 16628169 10.12968/bjon.2006.15.6.20681

[B41] Hughes DA Mlilligan A Mehta A Home therapy for lysosomal storage disorders Br J Nurs 2007 16 22 1386 1389 10.12968/bjon.2007.16.22.27768 18361386

[B42] Burton BK Guffon N Roberts J van de Ploeg AT Jones SA Home treatment with intravenous enzyme replacement therapy with idursulfase for mucopolysaccharidosis type II - data from the Hunter Outcome Survey Mol Genet Metab 2010 101 2-3 123 129 10.1016/j.ymgme.2010.06.011 20638311

[B43] Little C Gould R Hendriksz C The management of children with Hunter syndrome - a case study Br J Nurs 2009 18 5 321 322 19273994 10.12968/bjon.2009.18.5.40546

[B44] Bagewadi S Roberts J Mercer J Jones S Stephenson J Wraith JE Home treatment with Elaprase and Naglazyme is safe in patients with mucopolysaccharidoses types II and VI, respectively J Inherit Metab Dis 2008 31 6 733 737 10.1007/s10545-008-0980-0 18923918

[B45] Muenzer J Beck M Giugliani R Suzuki Y Tylki-Szymanska A Valayannopoulos V Vellodi A Wraith JE Idursulfase treatment of Hunter syndrome in children under 6 years old: results from the Hunter Outcome Survey Genet Med 2011 13 2 102 109 10.1097/GIM.0b013e318206786f 21233716

[B46] Vellodi A Wraith JE Cleary MA Ramaswami U Lavery C Jessop E Guidelines for the investigation and management of mucopolysaccharidosis type II Department of Health National Specialist Commissioning Group (NSCAG) 2007 http://www.dh.gov.uk/prod_consum_dh/groups/dh_digitalassets/@dh/@en/documents/digitalasset/dh_073340.pdf

[B47] Muenzer J Beck M Eng CM Escolar ML Giugliani R Guffon NH Harmatz P Kamin W Kampmann C Koseoglu ST Multidisciplinary management of Hunter syndrome Pediatrics 2009 124 6 e1228 1239 10.1542/peds.2008-0999 19901005

[B48] Aubourg P Blanche S Jambaque I Rocchiccioli F Kalifa G Naud-Saudreau C Rolland MO Debre M Chaussain JL Griscelli C Reversal of early neurologic and neuroradiologic manifestations of X-linked adrenoleukodystrophy by bone marrow transplantation N Engl J Med 1990 322 26 1860 1866 10.1056/NEJM199006283222607 2348839

[B49] Hobbs JR Hugh-Jones K Barrett AJ Byrom N Chambers D Henry K James DC Lucas CF Rogers TR Benson PF Reversal of clinical features of Hurler's disease and biochemical improvement after treatment by bone-marrow transplantation Lancet 1981 2 8249 709 712 6116856 10.1016/s0140-6736(81)91046-1

[B50] Krivit W Pierpont ME Ayaz K Tsai M Ramsay NK Kersey JH Weisdorf S Sibley R Snover D McGovern MM Bone-marrow transplantation in the Maroteaux-Lamy syndrome (mucopolysaccharidosis type VI). Biochemical and clinical status 24 months after transplantation N Engl J Med 1984 311 25 1606 1611 10.1056/NEJM198412203112504 6150438

[B51] Krivit W Shapiro E Kennedy W Lipton M Lockman L Smith S Summers CG Wenger DA Tsai MY Ramsay NK Treatment of late infantile metachromatic leukodystrophy by bone marrow transplantation N Engl J Med 1990 322 1 28 32 10.1056/NEJM199001043220106 1967188

[B52] Krivit W Shapiro EG Peters C Wagner JE Cornu G Kurtzberg J Wenger DA Kolodny EH Vanier MT Loes DJ Hematopoietic stem-cell transplantation in globoid-cell leukodystrophy N Engl J Med 1998 338 16 1119 1126 10.1056/NEJM199804163381605 9545360

[B53] Peters C Balthazor M Shapiro EG King RJ Kollman C Hegland JD Henslee-Downey J Trigg ME Cowan MJ Sanders J Outcome of unrelated donor bone marrow transplantation in 40 children with Hurler syndrome Blood 1996 87 11 4894 4902 8639864

[B54] Peters C Shapiro EG Anderson J Henslee-Downey PJ Klemperer MR Cowan MJ Saunders EF deAlarcon PA Twist C Nachman JB Hurler syndrome: II. Outcome of HLA-genotypically identical sibling and HLA-haploidentical related donor bone marrow transplantation in fifty-four children. The Storage Disease Collaborative Study Group Blood 1998 91 7 2601 2608 9516162

[B55] Priller J Flugel A Wehner T Boentert M Haas CA Prinz M Fernandez-Klett F Prass K Bechmann I de Boer BA Targeting gene-modified hematopoietic cells to the central nervous system: use of green fluorescent protein uncovers microglial engraftment Nat Med 2001 7 12 1356 1361 10.1038/nm1201-1356 11726978

[B56] Mullen CA Thompson JN Richard LA Chan KW Unrelated umbilical cord blood transplantation in infancy for mucopolysaccharidosis type IIB (Hunter syndrome) complicated by autoimmune hemolytic anemia Bone Marrow Transplant 2000 25 10 1093 1097 10.1038/sj.bmt.1702397 10828871

[B57] Young ID Harper PS Mild form of Hunter's syndrome: clinical delineation based on 31 cases Arch Dis Child 1982 57 11 828 836 10.1136/adc.57.11.828 6816147 PMC1628015

[B58] Bergstrom SK Quinn JJ Greenstein R Ascensao J Long-term follow-up of a patient transplanted for Hunter's disease type IIB: a case report and literature review Bone Marrow Transplant 1994 14 4 653 658 7858546

[B59] Coppa GV Gabrielli O Zampini L Pierani P Giorgi PL Jezequel AM Orlandi F Miniero R Busca A De Luca T Bone marrow transplantation in Hunter syndrome (mucopolysaccharidosis type II): two-year follow-up of the first Italian patient and review of the literature Pediatr Med Chir 1995 17 3 227 235 7567644

[B60] Hoogerbrugge PM Brouwer OF Bordigoni P Ringden O Kapaun P Ortega JJ O'Meara A Cornu G Souillet G Frappaz D Allogeneic bone marrow transplantation for lysosomal storage diseases. The European Group for Bone Marrow Transplantation Lancet 1995 345 8962 1398 1402 10.1016/S0140-6736(95)92597-X 7760610

[B61] Imaizumi M Gushi K Kurobane I Inoue S Suzuki J Koizumi Y Suzuki H Sato A Gotoh Y Haginoya K Long-term effects of bone marrow transplantation for inborn errors of metabolism: a study of four patients with lysosomal storage diseases Acta Paediatr Jpn 1994 36 1 30 36 8165905 10.1111/j.1442-200x.1994.tb03125.x

[B62] Resnick JM Krivit W Snover DC Kersey JH Ramsay NK Blazar BR Whitley CB Pathology of the liver in mucopolysaccharidosis: light and electron microscopic assessment before and after bone marrow transplantation Bone Marrow Transplant 1992 10 3 273 280 1330150

[B63] McKinnis EJ Sulzbacher S Rutledge JC Sanders J Scott CR Bone marrow transplantation in Hunter syndrome J Pediatr 1996 129 1 145 148 10.1016/S0022-3476(96)70202-0 8757575

[B64] Wynn RF Wraith JE Mercer J O'Meara A Tylee K Thornley M Church HJ Bigger BW Improved metabolic correction in patients with lysosomal storage disease treated with hematopoietic stem cell transplant compared with enzyme replacement therapy J Pediatr 2009 154 4 609 611 10.1016/j.jpeds.2008.11.005 19324223

[B65] Ito K Ochiai T Suzuki H Chin M Shichino H Mugishima H The effect of haematopoietic stem cell transplant on papules with 'pebbly' appearance in Hunter's syndrome Br J Dermatol 2004 151 1 207 211 10.1111/j.1365-2133.2004.05944.x 15270893

[B66] Araya K Sakai N Mohri I Kagitani-Shimono K Okinaga T Hashii Y Ohta H Nakamichi I Aozasa K Taniike M Localized donor cells in brain of a Hunter disease patient after cord blood stem cell transplantation Mol Genet Metab 2009 98 3 255 263 10.1016/j.ymgme.2009.05.006 19556155

[B67] Dickson P Novel treatments and future perspectives: outcomes of intrathecal drug delivery Int J Clin Pharmacol Ther 2009 47 Suppl 1 S124 127 20040323

[B68] Porto C Cardone M Fontana F Rossi B Tuzzi MR Tarallo A Barone MV Andria G Parenti G The pharmacological chaperone N-butyldeoxynojirimycin enhances enzyme replacement therapy in Pompe disease fibroblasts Mol Ther 2009 17 6 964 971 10.1038/mt.2009.53 19293774 PMC2835191

[B69] Cardone M Polito VA Pepe S Mann L D'Azzo A Auricchio A Ballabio A Cosma MP Correction of Hunter syndrome in the MPS II mouse model by AAV2/8-mediated gene delivery Hum Mol Genet 2006 15 7 1225 1236 10.1093/hmg/ddl038 16505002

[B70] Braun SE Aronovich EL Anderson RA Crotty PL McIvor RS Whitley CB Metabolic correction and cross-correction of mucopolysaccharidosis type II (Hunter syndrome) by retroviral-mediated gene transfer and expression of human iduronate-2-sulfatase Proc Natl Acad Sci USA 1993 90 24 11830 11834 10.1073/pnas.90.24.11830 8265633 PMC48078

[B71] Polito VA Cosma MP *IDS *crossing of the blood-brain barrier corrects CNS defects in MPS II mice Am J Hum Genet 2009 85 2 296 301 10.1016/j.ajhg.2009.07.011 19679226 PMC2725243

[B72] Friso A Tomanin R Salvalaio M Scarpa M Genistein reduces glycosaminoglycan levels in a mouse model of mucopolysaccharidosis type II Br J Pharmacol 2010 159 5 1082 1091 10.1111/j.1476-5381.2009.00565.x 20136838 PMC2839266

[B73] Hishitani T Wakita S Isoda T Katori T Ishizawa A Okada R Sudden death in Hunter syndrome caused by complete atrioventricular block J Pediatr 2000 136 2 268 269 10.1016/S0022-3476(00)70117-X 10657841

[B74] National Institute for Health and Clinical Excellence Prophylaxis against infective endocarditis. Antimicrobial prophylaxis against infective endocarditis in adults and children undergoing interventional procedures 2008 http://www.nice.org.uk/nicemedia/pdf/CG64NICEguidance.pdf 21656971

[B75] Dangel JH Cardiovascular changes in children with mucopolysaccharide storage diseases and related disorders: clinical and echocardiographic findings in 64 patients Eur J Pediatr 1998 157 7 534 538 10.1007/s004310050872 9686810

[B76] Bhattacharya K Gibson SC Pathi VL Mitral valve replacement for mitral stenosis secondary to Hunter's syndrome Ann Thorac Surg 2005 80 5 1911 1912 10.1016/j.athoracsur.2004.06.021 16242483

[B77] van Aerde J Plets C Van der Hauwaert L Hydrocephalus in Hunter Syndrome Acta Paediatr Belg 1981 34 2 93 96 6801919

[B78] Sheridan M Johnston I Hydrocephalus and pseudotumour cerebri in the mucopolysaccharidoses Childs Nerv Syst 1994 10 3 148 150 10.1007/BF00301079 8044807

[B79] Yatziv S Epstein CJ Hunter syndrome presenting as macrocephaly and hydrocephalus J Med Genet 1977 14 6 445 447 10.1136/jmg.14.6.445 146740 PMC1013642

[B80] Al Sawaf S Mayatepek E Hoffmann B Neurological findings in Hunter disease: pathology and possible therapeutic effects reviewed J Inherit Metab Dis 2008 31 4 473 480 10.1007/s10545-008-0878-x 18618289

[B81] Vinchon M Cotten A Clarisse J Chiki R Christiaens JL Cervical myelopathy secondary to Hunter syndrome in an adult Am J Neuroradiol 1995 16 7 1402 1403 7484623 PMC8338058

[B82] Ballenger CE Swift TR Leshner RT El Gammal TA McDonald TF Myelopathy in mucopolysaccharidosis type II (Hunter syndrome) Ann Neurol 1980 7 4 382 385 10.1002/ana.410070418 6769383

[B83] O'Brien DP Cowie RA Wraith JE Cervical decompression in mild mucopolysaccharidosis type II (Hunter syndrome) Childs Nerv Syst 1997 13 2 87 90 10.1007/s003810050049 9105743

[B84] Norman-Taylor F Fixsen JA Sharrard WJ Hunter's syndrome as a cause of childhood carpal tunnel syndrome: a report of three cases J Pediatr Orthop B 1995 4 1 106 109 10.1097/01202412-199504010-00018 7719824

[B85] Van Heest AE House J Krivit W Walker K Surgical treatment of carpal tunnel syndrome and trigger digits in children with mucopolysaccharide storage disorders J Hand Surg Am 1998 23 2 236 243 10.1016/S0363-5023(98)80120-2 9556262

[B86] Wraith JE Alani SM Carpal tunnel syndrome in the mucopolysaccharidoses and related disorders Arch Dis Child 1990 65 9 962 963 10.1136/adc.65.9.962 2121106 PMC1792093

[B87] Haddad FS Jones DH Vellodi A Kane N Pitt MC Carpal tunnel syndrome in the mucopolysaccharidoses and mucolipidoses J Bone Joint Surg Br 1997 79 4 576 582 10.1302/0301-620X.79B4.7547 9250742

[B88] Ashworth JL Biswas S Wraith E Lloyd IC Mucopolysaccharidoses and the eye Surv Ophthalmol 2006 51 1 1 17 10.1016/j.survophthal.2005.11.007 16414358

[B89] Link B de Camargo Pinto LL Giugliani R Wraith JE Guffon N Eich E Beck M Orthopedic manifestations in patients with mucopolysaccharidosis type II (Hunter syndrome) enrolled in the Hunter Outcome Survey (HOS) Orthopedic Reviews 2010 2 e16 56 64 10.4081/or.2010.e16 PMC3143973 21808707

[B90] Mendelsohn NJ Harmatz P Bodamer O Burton BK Giugliani R Jones SA Lampe C Malm G Steiner RD Parini R Importance of surgical history in diagnosing mucopolysaccharidosis type II (Hunter syndrome): data from the Hunter Outcome Survey Genet Med 2010 12 12 816 822 10.1097/GIM.0b013e3181f6e74d 21045710

[B91] Polgreen LE Miller BS Growth patterns and the use of growth hormone in the mucopolysaccharidoses J Pediatr Rehabil Med 2010 3 1 25 38 20563263 10.3233/PRM-2010-0106 PMC2886985

[B92] Richmond E Rogol AD Current indications for growth hormone therapy for children and adolescents Endocr Dev 2010 18 92 108 20523020 10.1159/000316130

[B93] Baxter L Bryant J Cave CB Milne R Recombinant growth hormone for children and adolescents with Turner syndrome Cochrane Database Syst Rev 2007 1 CD003887 10.1002/14651858.CD003887.pub2 17253498

[B94] Takeda A Cooper K Bird A Baxter L Frampton GK Gospodarevskaya E Welch K Bryant J Recombinant human growth hormone for the treatment of growth disorders in children: a systematic review and economic evaluation Health Technol Assess 2010 14 42 1 209 iii-iv 20849734 10.3310/hta14420

[B95] Docquier PL Mousny M Jouret M Bastin C Rombouts JJ Orthopaedic concerns in children with growth hormone therapy Acta Orthop Belg 2004 70 4 299 305 15481411

[B96] Polgreen LE Plog M Schwender JD Tolar J Thomas W Orchard PJ Miller BS Petryk A Short-term growth hormone treatment in children with Hurler syndrome after hematopoietic cell transplantation Bone Marrow Transplant 2009 44 5 279 285 10.1038/bmt.2009.31 19252529 PMC3071029

[B97] Simmons MA Bruce IA Penney S Wraith E Rothera MP Otorhinolaryngological manifestations of the mucopolysaccharidoses Int J Pediatr Otorhinolaryngol 2005 69 5 589 595 10.1016/j.ijporl.2005.01.017 15850680

[B98] Kamin W Diagnosis and management of respiratory involvement in Hunter syndrome Acta Paediatr 2008 97 Suppl 457 57 60 10.1111/j.1651-2227.2008.00650.x 18339190

[B99] Ginzburg AS Onal E Aronson RM Schild JA Mafee MF Lopata M Successful use of nasal-CPAP for obstructive sleep apnea in Hunter syndrome with diffuse airway involvement Chest 1990 97 6 1496 1498 10.1378/chest.97.6.1496 2112082

[B100] Jeong HS Cho DY Ahn KM Jin DK Complications of tracheotomy in patients with mucopolysaccharidoses type II (Hunter syndrome) Int J Pediatr Otorhinolaryngol 2006 70 10 1765 1769 10.1016/j.ijporl.2006.05.021 16831472

[B101] Walker RW Allen DL Rothera MR A fibreoptic intubation technique for children with mucopolysaccharidoses using the laryngeal mask airway Paediatr Anaesth 1997 7 5 421 426 10.1046/j.1460-9592.1997.d01-102.x 9308068

[B102] Walker RW The laryngeal mask airway in the difficult paediatric airway: an assessment of positioning and use in fibreoptic intubation Paediatr Anaesth 2000 10 1 53 58 10.1046/j.1460-9592.2000.00425.x 10632910

[B103] Walker RW Darowski M Morris P Wraith JE Anaesthesia and mucopolysaccharidoses. A review of airway problems in children Anaesthesia 1994 49 12 1078 1084 10.1111/j.1365-2044.1994.tb04360.x 7864325

[B104] Moores C Rogers JG McKenzie IM Brown TC Anaesthesia for children with mucopolysaccharidoses Anaesth Intensive Care 1996 24 4 459 463 8862643 10.1177/0310057X9602400408

[B105] Hopkins R Watson JA Jones JH Walker M Two cases of Hunter's syndrome: the anaesthetic and operative difficulties in oral surgery Br J Oral Surg 1973 10 3 286 299 4198586 10.1016/s0007-117x(72)80058-1

[B106] Walker RW Colovic V Robinson DN Dearlove OR Postobstructive pulmonary oedema during anaesthesia in children with mucopolysaccharidoses Paediatr Anaesth 2003 13 5 441 447 10.1046/j.1460-9592.2003.00969.x 12791120

[B107] The Society for Mucopolysaccharide Diseases http://www.mpssociety.co.uk

[B108] Martin HR Poe MD Reinhartsen D Pretzel RE Roush J Rosenberg A Dusing SC Escolar ML Methods for assessing neurodevelopment in lysosomal storage diseases and related disorders: a multidisciplinary perspective Acta Paediatr 2008 97 Suppl 457 69 75 10.1111/j.1651-2227.2008.00651.x 18339192

[B109] Man TT Tsai PS Rau RH Cheng CR Ko YP Wu KH Children with mucopolysaccharidoses: three cases report Acta Anaesthesiol Sin 1999 37 2 93 96 10410410

[B110] Rovers MM Balemans WA Sanders EA van der Ent CK Zielhuis GA Schilder AG Persistence of upper respiratory tract infections in a cohort followed from childhood to adulthood Fam Pract 2006 23 3 286 290 10.1093/fampra/cml001 16517546

[B111] Kværner KJ Nafstad P Jaakkola JJ Upper respiratory morbidity in preschool children: a cross-sectional study Arch Otolaryngol Head Neck Surg 2000 126 10 1201 1206 11031406 10.1001/archotol.126.10.1201

[B112] Young ID Harper PS The natural history of the severe form of Hunter's syndrome: a study based on 52 cases Dev Med Child Neurol 1983 25 4 481 489 6413286 10.1111/j.1469-8749.1983.tb13794.x

[B113] Downs AT Crisp T Ferretti G Hunter's syndrome and oral manifestations: a review Pediatr Dent 1995 17 2 98 100 7603911

[B114] Brama I Gay I Feinmesser R Springer C Upper airway obstruction in Hunter syndrome Int J Pediatr Otorhinolaryngol 1986 11 3 229 235 10.1016/S0165-5876(86)80034-9 3095256

[B115] Shih SL Lee YJ Lin SP Sheu CY Blickman JG Airway changes in children with mucopolysaccharidoses Acta Radiol 2002 43 1 40 43 11972460 10.1080/028418502127347628

[B116] Shapiro J Strome M Crocker AC Airway obstruction and sleep apnea in Hurler and Hunter syndromes Ann Otol Rhinol Laryngol 1985 94 5 I 458 461 3931528 10.1177/000348948509400508

[B117] Orliaguet O Pepin JL Veale D Kelkel E Pinel N Levy P Hunter's syndrome and associated sleep apnoea cured by CPAP and surgery Eur Respir J 1999 13 5 1195 1197 10.1034/j.1399-3003.1999.13e42.x 10414426

[B118] Leighton SE Papsin B Vellodi A Dinwiddie R Lane R Disordered breathing during sleep in patients with mucopolysaccharidoses Int J Pediatr Otorhinolaryngol 2001 58 2 127 138 10.1016/S0165-5876(01)00417-7 11278021

[B119] Morehead JM Parsons DS Tracheobronchomalacia in Hunter's syndrome Int J Pediatr Otorhinolaryngol 1993 26 3 255 261 10.1016/0165-5876(93)90096-L 8509249

[B120] Thappa DM Singh A Jaisankar TJ Rao R Ratnakar C Pebbling of the skin: a marker of Hunter's syndrome Pediatr Dermatol 1998 15 5 370 373 10.1046/j.1525-1470.1998.1998015370.x 9796587

[B121] Benson PF Button LR Fensom AH Dean MF Lumbar kyphosis in Hunter's disease (MPS II) Clin Genet 1979 16 5 317 322 117962 10.1111/j.1399-0004.1979.tb01009.x

[B122] Van Meir N De Smet L Carpal tunnel syndrome in children Acta Orthop Belg 2003 69 5 387 395 14648946

[B123] Parsons VJ Hughes DG Wraith JE Magnetic resonance imaging of the brain, neck and cervical spine in mild Hunter's syndrome (mucopolysaccharidoses type II) Clin Radiol 1996 51 10 719 723 10.1016/S0009-9260(96)80246-7 8893643

[B124] Vieira T Schwartz I Muñoz V Pinto L Steiner C Ribeiro M Boy R Ferraz V de Paula A Kim C Mucopolysaccharidoses in Brazil: what happens from birth to biochemical diagnosis? Am J Med Genet A 2008 146A 13 1741 1747 10.1002/ajmg.a.32320 18546277

[B125] François J Ocular manifestations of the mucopolysaccharidoses Ophthalmologica 1974 169 5 345 361 10.1159/000307137 4370235

[B126] Gills JP Hobson R Hanley WB McKusick VA Electroretinography and fundus oculi findings in Hurler's disease and allied mucopolysaccharidoses Arch Ophthalmol 1965 74 5 596 603 4954407 10.1001/archopht.1965.00970040598003

[B127] Collins ML Traboulsi EI Maumenee IH Optic nerve head swelling and optic atrophy in the systemic mucopolysaccharidoses Ophthalmology 1990 97 11 1445 1449 2123975 10.1016/s0161-6420(90)32400-4

[B128] Yoskovitch A Tewfik TL Brouillette RT Schloss MD Der Kaloustian VM Acute airway obstruction in Hunter syndrome Int J Pediatr Otorhinolaryngol 1998 44 3 273 278 10.1016/S0165-5876(98)00063-9 9780074

[B129] Linhart A Elliott PM The heart in Anderson-Fabry disease and other lysosomal storage disorders Heart 2007 93 4 528 535 10.1136/hrt.2005.063818 17401074 PMC1861503

